# Prevalence and Characteristics of Telehealth Utilization in the United States

**DOI:** 10.1001/jamanetworkopen.2020.22302

**Published:** 2020-10-26

**Authors:** Shira H. Fischer, Kristin N. Ray, Ateev Mehrotra, Erika Litvin Bloom, Lori Uscher-Pines

**Affiliations:** 1 RAND Corporation, Boston, Massachusetts; 2 Health Policy Institute, UPMC Community Pediatrics, University of Pittsburgh, Pennsylvania; 3 Department of Health Care Policy, Harvard Medical School, Boston, Massachusetts; 4 RAND Corporation, Arlington, Virginia

## Abstract

**Importance:**

Telehealth services, which allow patients to communicate with a remotely located clinician, are increasingly available; however, prevalence of telehealth use, including videoconferencing visits, remains unclear.

**Objective:**

To measure the use of and willingness to use telehealth modalities across the US population.

**Design, Setting, and Participants:**

This survey study, conducted between February 2019 and April 2019, asked participants about their use of different telehealth modalities, reasons for not using videoconferencing visits, and willingness to use videoconferencing visits. Questions were continuously posed to panel members and closed after 2555 responses were obtained, at which point 3932 panel members had been invited, for a 65.0% response rate.

**Exposures:**

Demographic characteristics (ie, age, sex, race, rural/urban residency, education level, and income).

**Main Outcomes and Measures:**

Self-reported use of specific telehealth modalities, reasons for nonuse, and willingness to use videoconferencing in the future.

**Results:**

A total of 2555 individuals completed the survey with a mean (SD) age of 57.2 (14.2) years; 1453 respondents (weighted percentage, 51.9%) were women, and 2043 (weighted percentage, 73.4%) were White individuals. Overall, 1343 respondents (weighted percentage, 50.8%) reported use of a nontelephone telehealth modality, ranging from 873 respondents (weighted percentage, 31.9%) for patient portals and 89 respondents (weighted percentage, 4.2%) for videoconferencing visits. Although 1309 respondents (weighted percentage, 49.2%) overall answered that they were willing or very willing to use videoconferencing visits, respondents who were Black individuals (OR, 0.58; 95% CI, 0.38-0.91), aged older than 65 years (OR, 0.51; 95% CI, 0.40-0.66), or had less education (high school or less vs advanced degrees: OR, 0.37; 95% CI, 0.25-0.56) were less likely to express willingness.

**Conclusions and Relevance:**

Despite the focused policy attention on videoconferencing visits, the results of this survey study suggest that other forms of telehealth were more dominant prior to 2020. Targeted efforts may be necessary for videoconferencing visits to reach patient groups who are older or have less education, and payer policies supporting other forms of telemedicine may be appropriate to enhance access.

## Key Points

**Question:**

What is the prevalence of telehealth use in the US, and what demographic characteristics are associated with it?

**Findings:**

This survey study of 2555 US adult respondents in an internet-based representative panel about use of telehealth found that only approximately 4% of participants had engaged in videoconferencing visits. While 49% of respondents expressed interest in using videoconferencing in the future, those who were older, identified as Black individuals, or reported lower levels of education expressed less willingness to use videoconferencing.

**Meaning:**

The findings of this study suggest further efforts may be needed to enhance use of and access to videoconferencing visits among the US population.

## Teaser

This survey study of participants in an online panel examined willingness to participate in telehealth services to access health services with remote clinicians among participants categorized by sex, age, race/ethnicity, income, and education level.

## Introduction

In 2020, tasks that once required traveling to a physical location are increasingly accomplished from home with a smartphone, including ordering groceries and depositing checks.^1^ The use of personal electronic devices to improve access and convenience has spread to health care. Various forms of telehealth services, including text messaging, email, patient portals, videoconferencing visits, and e-visits are becoming increasingly available in the US,^2^ and have the potential to increase access to care and reduce health disparities among rural and underserved populations.^3,4^ However, until the coronavirus disease 2019 (COVID-19) pandemic began in March 2020, use of telehealth was relatively rare, and estimates of telehealth adoption in the US were variable and had numerous limitations.^5,6,7,8,9^ For example, studies commonly focused on only 1 form of telehealth, used inconsistent definitions of telehealth, and/or engaged small, nonrepresentative samples. Also, commonly cited statistics on telehealth utilization usually came from market research studies sponsored by organizations with a financial interest in suggesting telehealth use is high. To characterize the current increase in telehealth adoption spurred by the pandemic, it is important to understand baseline utilization.

To address the limitations of existing research studies, we conducted a cross-sectional survey between February 2019 and April 2019 of the general public to estimate the use of different forms of telehealth and reasons for nonuse. We emphasized questions on use of videoconferencing visits because this modality has been the focus of policy attention in recent years and is most consistently included in different organizations’ definitions of telehealth.

## Methods

The RAND American Life Panel (ALP) is an internet panel recruited primarily using probability sampling methods. The ALP consists of approximately 6000 active respondents aged 18 years and older and allows for nationally representative estimates after weighting. Respondents use their own internet-connected devices or devices that RAND provides to complete surveys, and they receive incentives for each completed survey that vary based on survey length. The ALP has been used extensively in prior research.^10,11^ Collection of survey data through ALP was deemed to fall under the adult interview and survey exemption from informed consent by the institutional review board at RAND. Data for this study were collected in a manner consistent with the American Association for Public Opinion Research (AAPOR) reporting guideline.

Following an environmental scan of existing surveys^5,6,7,8,9^ and their instruments, where available, we developed 4 telehealth-related questions gathering data on (1) use of different telehealth modalities at any point in the past; (2) use of telephone or video conferencing visits in the past year and whether the visit(s) was with a familiar or unfamiliar physician; (3) reasons for not using videoconferencing visits; and (4) willingness to use videoconferencing visits (using a 5-point Likert scale). Survey questions are included in the eAppendix in the Supplement. We conducted cognitive testing on the questions with 6 volunteers who had varying levels of experience with telehealth. These questions were then fielded as an independent module within a longer cross-sectional survey, and responses to these questions were merged with dozens of demographic variables maintained by the ALP for all active panelists. Individual respondents were invited to complete the survey until a sufficient number of responses were collected, with the overall goal of 2500 responses.

In our analysis, we focused on nontelephone telehealth modalities that have only become more widespread in health care in the past decade, such as e-visits, videoconferencing visits, patient portals, and email. In the survey, we asked specifically about use of these modalities to communicate with a physician to get advice about a health issue, to exclude routine interactions during which medical advice is not generally provided (eg, telephone calls to schedule appointments or refill medications). We examined differences in responses by demographic characteristics, including sex, age group, race, income, location (urban vs rural), education level, and region of the US.

### Statistical Analysis

Pearson χ^2^ tests with Yates continuity corrections and survey weighting were used for bivariate comparisons. To identify characteristics associated with willingness to participate in videoconferencing visits, we used a weighted regression model. Willingness to participate was treated as a binary outcome, with participants who described themselves as willing or very willing to use videoconferencing visits considered willing and participants who described themselves as neutral, unwilling, or very unwilling considered unwilling. We adjusted for sex, race, age, urban/rural location, region, income, and education. Missing data were limited (<10% of all variables) and likely random; entries with missing values were dropped from the analysis. We assessed multivariable regression models for collinearity.

Weighting was used to adjust the results from a random sample to match the population composition. Survey weights, which were developed using a raking method to match population distributions retrieved from the Current Population Survey, were constructed for the respondent sample to generate nationally representative estimates.^12,13^

Analyses were conducted in R version 3.5.1 (R Project for Statistical Computing). The threshold for statistical significance was *P* < .05 in 2-sided tests (where available, with continuity correction).

## Results

Overall, 3932 panelists were invited and 2555 (65.0%) completed the survey with a mean (SD) age of 57.2 (14.2) years (after applying sampling weights, the mean [SE] age of respondents was 49.6 [0.6] years). A total of 1453 (weighted percentage, 51.9%) were women, and 2043 (weighted percentage, 73.4%) were White individuals; 1301 respondents (weighted percentage, 34.4%) had a bachelor’s degree or higher and 2010 (weighted percentage, 80.6%) resided in urban counties. Respondent characteristics are described in Table 1.

**Table 1.** Respondent Characteristics, Weighted and Unweighted

Characteristic	Respondents, No. (%)^a^	
	Unweighted (n = 2555)	Weighted
Age, y		
Mean	57.2 (SD, 14.2)	49.6 (SE, 0.60)
Median (range)	59.0 (22.0-90.0)	NA
21-40	405 (15.9)	NA
41-65	1382 (54.1)	NA
>65	768 (30.1)	NA
Missing	5 (0.2)	NA
Sex		
Women	1453 (56.9)	1325 (51.9)
Men	1102 (43.1)	1230 (48.1)
Race		
White	2043 (80.0)	1875 (73.4)
Black/African American	262 (10.3)	321 (12.6)
American Indian or Alaskan Native	30 (1.2)	60 (2.3)
Asian American or Pacific Islander	71 (2.8)	74 (2.9)
Other	149 (5.8)	225 (8.8)
Education		
High school or less	358 (14.0)	989 (38.7)
Associate degree or some college	896 (35.1)	686 (26.9)
Bachelor’s degree	686 (26.8)	473 (18.5)
Advanced degree	615 (24.1)	407 (15.9)
Location		
Rural or small town, population <50 000	542 (21.2)	496 (19.4)
Small to midsize city or large city, population ≥50 000	2010 (78.7)	2056 (80.6)
Missing	3 (0.1)	NA
Region		
Midwest	467 (18.3)	400 (15.7)
Northeast	883 (34.6)	925 (36.2)
South	472 (18.5)	485 (19.0)
West	732 (28.6)	745 (29.2)
Missing	1 (0.0)	NA
Family income, $		
Highest income, ≥200 000	145 (5.7)	130 (5.1)
High income, 125 000 up to 200 000	302 (11.8)	292 (11.4)
Middle class, 50 000 up to 125 000	1114 (43.6)	1090 (42.7)
Low income, 20 000 up to 50 000	697 (27.3)	661 (25.9)
Lowest income, <20 000	293 (11.5)	380 (14.9)
Missing	4 (0.2)	NA

Abbreviation: NA, not applicable.

^a^ Percentages may not add up to 100% because of rounding.

### Use of Different Telehealth Modalities

**Figure 1.**
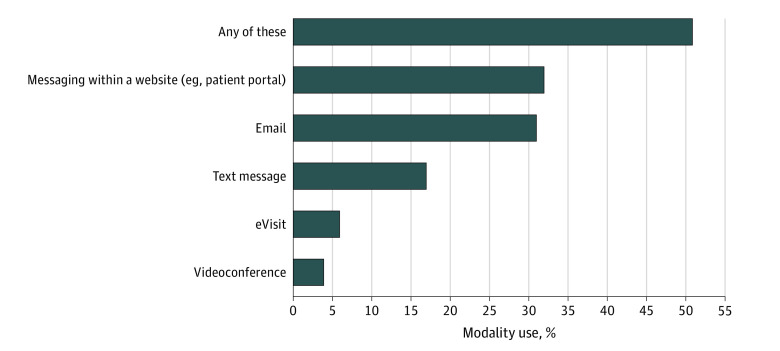
Proportion of Respondents Who Reported Ever Using Various Telehealth Modalities, Weighted Multiple choices could be selected; telephone visits were not included in this analysis. In this survey, an eVisit was described as “when you answer questions about your health issue online and hear back from a doctor later.”

In total, 1343 individuals (weighted percentage, 50.8%) reported use of at least 1 telehealth modality (not including telephone) to communicate with a physician to get advice about a health issue. Among the newer (ie, nontelephone) modalities, use was highest for patient portals (873 respondents; weighted percentage, 31.9%), followed by email (797 respondents; weighted percentage, 30.5%), text messaging (379 respondents; weighted percentage, 16.7%), e-visits (153 respondents; weighted percentage, 6.3%), and videoconferencing visits (89 respondents; weighted percentage 4.2%) (Figure 1). When telephone calls are included as a telehealth strategy, a total of 2157 respondents (weighted percentage, 83.0%) reported that they had used 1 or more telehealth modalities (telephone only: 814 respondents; weighted percentage, 32.2%). Among those who had videoconferencing visits in the past year, more than half (54.5%) were with an unfamiliar physician (vs a respondent’s usual physician).

Men (567 respondents; weighted percentage, 47.5%) were significantly less likely than women (776 respondents; weighted percentage, 53.8%) to report use of a nontelephone telehealth modality (*P* = .001). Regional differences were also significant, with lower use in the South (226 respondents; weighted percentage, 47.4%), Midwest (244 respondents; weighted percentage, 48.5%), and Northeast (452 respondents; weighted percentage, 48.7%) compared with use in the West (420 respondents; weighted percentage, 56.7%; *P* = .002).

Focusing more specifically on videoconference visits, there was a significantly lower rate of use among those older than age 65 years vs those 65 years or younger (13 respondents; weighted percentage, 1.8% vs 76 respondents; weighted percentage, 4.8%; *P* = .004), and there was a lower rate of use among those with a high school education or less vs among those with advanced degrees (12 respondents; weighted percentage, 2.7% vs 17 respondents; weighted percentage, 5.5%; *P* = .02 for comparison among all educational groups). There were no differences for sex, race, urban/rural location, or income.

### Willingness to Use Videoconferencing Visits

**Figure 2.**
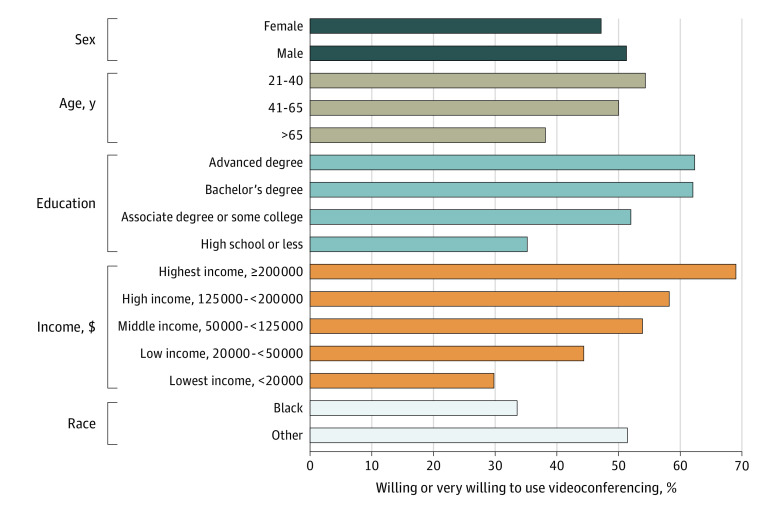
Univariate Comparisons of Willingness to Use Videoconferencing by Demographic Characteristic Comparison for sex *P* < .05; all other comparisons *P* < .001.

Across all respondents, including those who did and did not report participating in videoconferencing visits, 1309 respondents (weighted percentage, 49.2%) reported that they were willing or very willing to participate in a videoconferencing visit. In univariate analyses, adults older than 65 years were much less likely than those who were younger (≤65 years) to be willing to use this modality (335 respondents; weighted percentage, 38.2% vs 974 respondents; weighted percentage, 51.9%; *P* < .001). Respondents who were Black individuals and respondents living below or near the federal poverty level were also less likely to report willingness to use videoconferencing. Compared with other racial/ethnic groups, 33.6% of Black respondents reported willingness to use videoconferencing vs 51.5% of respondents from other races (*P* < .001). In addition, 69.1% of respondents (unweighted total, 103) with incomes of over $200 000 reported willingness to use videoconferencing vs 29.9% (118) with incomes of less than $20 000 and 44.4% (309) with incomes between $20 000 and $50 000 (*P* < .001). Results are shown in Figure 2.

In multivariable weighted quasibinomial regression models adjusting for race, age, urban/rural location, US region, income, and education, respondents older than 65 years were less likely to report willingness to participate in videoconferencing visits compared with those who were younger, and Black respondents also had reduced odds of reporting willingness to participate in videoconferencing visits compared with non-Black participants (age >65 years: OR, 0.51; 95% CI, 0.40-0.66; Black: OR, 0.58; 95% CI, 0.38-0.91). We also saw differences in education: those with a high school education or less, compared with those with advanced degrees, were less likely to be willing or very willing to participate in videoconferencing (OR, 0.37; 95% CI, 0.25-0.56).

Sex, region, urban location, and income were not associated with significant differences in willingness in this model. The full multivariable analysis results can be found in Table 2.

**Table 2.** Willingness to Use Telehealth by Demographic Characteristic (Sex, Age, Race, Income, Education): Multivariable Regression

Category	Odds ratio (95% CI)	SE	t value	Probability > t	P value
Total	0.48 (0.32-0.73)	0.21	−3.44	<0.001	<.001
Sex					
Women	1 [Reference]	NA	NA	0.31	NA
Men	1.15 (0.88-1.51)	0.14	1.02		
Race					
Other^a^	1 [Reference]	NA	NA	0.02	NA
Black	0.59 (0.38-0.91)	0.22	−2.36		
Age, y					
≤65	1 [Reference]	NA	NA	NA	<.001
>65	0.51 (0.40-0.66)	0.13	−5.30	<0.001	
Urban/Rural					
Rural	1 [Reference]	NA	NA	0.40	NA
Urban	1.14 (0.84-1.55)	0.16	0.83		
Region					
Northeast	1 [Reference]	NA	NA	NA	NA
Midwest	0.90 (0.64-1.27)	0.18	−0.59	0.55	
South	0.79 (0.56-1.12)	0.18	−1.31	0.19	
West	1.31 (0.91-1.88)	0.18	1.46	0.14	
Income, $					
<50 000	1 [Reference]	NA	NA	0.07	NA
≥50 000	1.34 (0.98-1.83)	0.16	1.84		
Education					
High school or less	1 [Reference]	NA	NA	NA	<.001
Associate degree or some college	1.98 (1.39-2.83)	0.18	3.77	<0.001	
Bachelor’s degree	2.65 (1.79-3.90)	0.20	4.91	<0.001	
Advanced degree	2.67 (1.79-3.99)	0.20	4.82	<0.001	

Abbreviation: NA, not applicable.

^a^ Racial groups included in this category were White/Caucasian, American Indian or Alaskan Native, Asian or Pacific Islander, and Other.

### Reasons for Not Using Videoconferencing Visits

**Figure 3.**
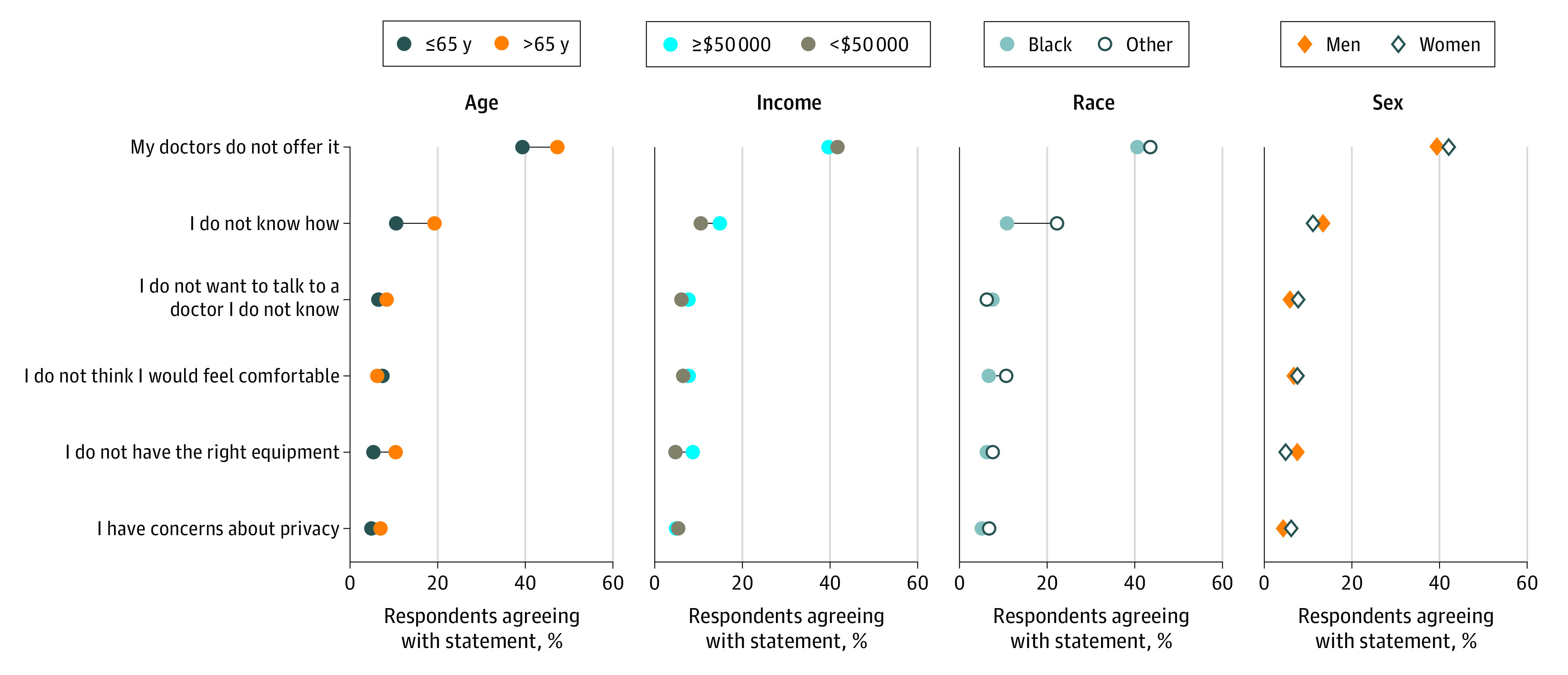
Reasons for Not Using Videoconferencing by Demographic Group Respondents could answer with more than 1 reason.

Respondents who had not participated in videoconferencing visits during the past year indicated up to 3 reasons for nonuse (Figure 3). The most frequently selected reasons were no perceived need (1161 respondents; weighted percentage, 47.3%), that their physician does not offer it (1092 respondents; weighted percentage, 40.9%), not knowing how (313 respondents; weighted percentage, 12.1%), and not feeling comfortable (192 respondents; weighted percentage, 7.0%).

Older adults (aged >65 years) were more likely to identify the fact that their physician does not offer videoconferencing visits as a barrier (367 respondents; weighted percentage, 47.3%) compared with younger adults (725 respondents; weighted percentage, 39.3%; *P* = .001). Older adults were also significantly more likely (147 respondents; weighted percentage, 19.2%) to report that they did not know how to use videoconferencing compared with respondents who were younger (age 21-40 years: 30 respondents; weighted percentage, 8.8%; *P* < .001). Similarly, those with the lowest incomes (<$20 000: 49 respondents; weighted percentage, 19.5%) were more likely to say they did not know how compared with those with the highest incomes (≥$200 000: 11 respondents; weighted percentage, 5.4%; *P* < .001).

Compared with participants belonging to other races, Black respondents were significantly less likely to endorse that they did not have the need (91 respondents; weighted percentage, 26.9% vs 1070 respondents; weighted percentage, 50.3%; *P* < .001) and more likely to report that they did not know how to use videoconferencing visits (34 respondents; weighted percentage, 22.1% vs 279 respondents; weighted percentage, 10.6%; *P* < .001). There were no differences by race in identification of physicians offering videoconferencing visits as a barrier.

## Discussion

In a nationally representative survey of US residents, we found that just more than half of respondents had ever used a nontelephone telehealth modality to discuss a health issue with a physician. Despite the focus of policy debates on videoconferencing visits, our data suggest that the typical US resident is most familiar with other forms of telehealth, including patient portals, email, and text messaging. Efforts to expand reimbursement, remove regulatory barriers, and directly incentivize use of videoconferencing visits had not facilitated widespread use as of 2019. Our findings also suggest the need to evaluate and improve these other modalities that patients are using to interact directly with physicians and to ensure that use of these modalities is appropriately incentivized for physicians.

Although our data are more recent than many efforts to assess telehealth adoption, we found lower rates of use of videoconferencing visits (vs an approximate range of 4%-22% in other studies) and less willingness to use it compared with other general population surveys and industry polls.^7,8,9,14^ Differences in findings may be attributable in part to how samples are constructed. For example, a nationally representative survey conducted in 2015 to 2016 by the Association of American Medical Colleges only included adults who had a need for medical care in the past 12 months.^9^

In our representative sample of US adults, nearly half of respondents were willing to use videoconferencing visits, but Black and older individuals as well as individuals with less education were both less likely and less willing to use videoconferencing visits. This is concerning because African American individuals, rural residents, and individuals with lower socioeconomic status have been shown to have decreased access to care.^15^ Disparities in use and willingness to use telehealth are critical to identify because telemedicine advocates often cite telehealth’s ability to improve access and address disparities in utilization of health services. However, if the populations who would most benefit from it are the least likely to use it, simply offering telehealth to a population may increase disparities in access.

### Limitations and Strengths

This study had limitations. The ALP is an internet panel, and even though internet access is provided to offline households, it faces the limitations of all probability-based internet panels.^16^ However, a strength of our study was that it captured a nationally representative sample of the general public, including offline households. Our response rate was 65%, which is relatively high for online surveys; however, we do not know if those who did not respond were significantly different than those who did.

## Conclusions

During the COVID-19 pandemic, which began in 2020, expanded reimbursement for telehealth quickly increased telehealth utilization; however, it is unclear whether high utilization rates will be maintained, especially if temporary policies to facilitate the provision of telehealth services are rolled back.^17^ Regardless of whether longstanding reimbursement and regulatory barriers to telehealth return after the emergency declaration ends, our findings suggest that targeted efforts to address awareness, integration with primary care, and ease of use may be necessary to reach African American individuals, rural, and lower-income patients to ensure they have equitable access to health care delivery innovations.

## Notes

Supplement. **eAppendix.** Questionnaire
