# Supplementary material for: Prevalence and Characteristics of Telehealth Utilization in the United States
Source: JAMA Netw Open. 2020 Oct 26;3(10):e2022302. doi: 10.1001/jamanetworkopen.2020.22302 (PMC7588937; doi:10.1001/jamanetworkopen.2020.22302)
Supplement: Supplement. — eAppendix. Questionnaire [file jamanetwopen-e2022302-s001.pdf]

Supplemental Online Content

Fischer SH, Ray KN, Mehrotra A, Bloom EL, Uscher-Pines L. Prevalence and characteristics of telehealth utilization in the United States. *JAMA Netw Open*. 2020;3(10):e2022302. doi:10.1001/jamanetworkopen.2020.22302

eAppendix. Questionnaire

This supplemental material has been provided by the authors to give readers additional information about their work.

© 2020 Fischer SH et al. *JAMA Network Open.*

eAppendix. Questionnaire

Introduction: With virtual healthcare, you can use a computer or smartphone to communicate with a doctor about a health issue. Two common types of virtual healthcare visits are telephone and videoconferencing visits. With a telephone visit, you can get advice about a health issue by telephone. With a videoconferencing visit, you interact with a doctor over video, and it works like Skype or FaceTime. Both kinds of visits can be with a personal doctor who you also see in-person OR with an unfamiliar doctor you have never seen in-person. The following questions ask about your experiences with virtual healthcare.

Q1: Select all the ways you have **ever** communicated with a doctor to get advice about a health issue **(Select all that apply)**

1. Text message
2. Email
3. Messaging within a website (e.g., patient portal)
4. Telephone
5. Videoconference
6. eVisit (when you answer questions about your health issue online and hear back from a doctor later)
7. None of these

Q2: In the past 12 months, have you had a telephone or videoconferencing visit with a doctor to get advice about a health issue? **(Select all that apply)**

1. Yes, telephone visit with my personal doctor
2. Yes, telephone visit with an unfamiliar doctor
3. Yes, videoconferencing visit with my personal doctor
4. Yes, videoconferencing visit with an unfamiliar doctor
5. None of these

Q3: [For respondents who did not answer yes to videoconferencing Q2:] What are the main reasons you have not participated in a videoconferencing visit with a doctor in the last 12 months? (**Select up to 3)**

1. Too costly/not covered by my health insurance
2. My doctors don't offer it
3. I don't want to talk to a doctor I don't know
4. I don't think I'd get good quality care
5. I don't think I'd feel comfortable
6. Too hard to communicate by video
7. I have concerns about privacy

© 2020 Fischer SH et al. *JAMA Network Open.*

8. I don’t have the right equipment
9. I don’t know how
10. I have not had the need (e.g., I have not been sick)
11. Other, please specify________________________

Q4: On a 1-5 scale, rate your willingness to participate in a videoconferencing visit with a doctor for a non-emergency health issue …

1 2 3 4 5

© 2020 Fischer SH et al. *JAMA Network Open.*
